# The impact of a community-led program promoting weight loss and healthy living in Aboriginal communities: the New South Wales Knockout Health Challenge

**DOI:** 10.1186/s12889-017-4955-7

**Published:** 2017-12-13

**Authors:** Erin Passmore, Brooke Shepherd, Andrew Milat, Louise Maher, Kiel Hennessey, Rachael Havrlant, Michelle Maxwell, Wendy Hodge, Fiona Christian, Justin Richards, Jo Mitchell

**Affiliations:** 1 NSW Ministry of Health, North Sydney, NSW Australia; 2 Sydney School of Public Health, University of Sydney, Sydney, NSW Australia; 3 NSW Agency for Clinical Innovation, Chatswood, NSW Australia; 4 ARTD Consultants, Level 4/352 Kent Street, Sydney, NSW Australia; 5 Charles Perkins Centre, University of Sydney, Sydney, NSW Australia

**Keywords:** Aboriginal, Obesity and overweight, Weight loss, Physical activity, Lifestyle program, Health promotion, Program evaluation

## Abstract

**Background:**

Aboriginal people in Australia experience significant health burden from chronic disease. There has been limited research to identify effective healthy lifestyle programs to address risk factors for chronic disease among Aboriginal people.

**Methods:**

The Knockout Health Challenge is a community-led healthy lifestyle program for Aboriginal communities across New South Wales, Australia. An evaluation of the 2013 Knockout Health Challenge was undertaken. Participants’ self-reported physical activity and diet were measured at four time points - at the start and end of the Challenge (via paper form), and 5 and 9 months after the Challenge (via telephone survey). Participants’ weight was measured objectively at the start and end of the Challenge, and self-reported (via telephone survey) 5 and 9 months after the Challenge. Changes in body composition, physical activity and diet between time points were analysed using linear mixed models. As part of the telephone survey participants were also asked to identify other impacts of the Challenge; these were analysed descriptively (quantitative items) and thematically (qualitative items).

**Results:**

A total of 586 people registered in 22 teams to participate in the Challenge. The mean weight at the start was 98.54kg (SD 22.4), and 94% of participants were overweight or obese. Among participants who provided data at all four time points (*n*=122), the mean weight loss from the start to the end of the Challenge was 2.3kg (95%CI -3.0 to -1.9, *p*<0.001), and from the start to 9 months after the Challenge was 2.3kg (95%CI -3.3 to -1.3, *p*<0.001). Body mass index decreased by an average of 0.9kg/m^2^ (95%CI -1.0 to -0.7, *p*<0.001) from the start to the end of the Challenge, and 0.8kg/m^2^ (95%CI -1.2 to -0.4, *p*<0.001) 9 months after. At the end of the Challenge, participants reported they were more physically active and had increased fruit and vegetable consumption compared with the start of the Challenge, and identified a range of other positive impacts.

**Conclusions:**

The Challenge was effective in reducing weight and promoting healthy lifestyles among Aboriginal people across New South Wales, and has potential to contribute to closing the health gap between Aboriginal and non-Aboriginal people.

## Background

There are large disparities in the health status of Aboriginal people compared with non-Aboriginal people in Australia. Life expectancy is approximately 10.6 years lower for Aboriginal males and 9.5 years lower for Aboriginal females, compared with the non-Aboriginal population [1]. Chronic diseases account for two-thirds of this health gap: circulatory disease (24% of the gap), endocrine, metabolic and nutritional disorders (21%), cancer (12%), and respiratory diseases (12%) [1] . Aboriginal people also have a high prevalence of risk factors for chronic disease. For example, 66% of Aboriginal adults are overweight or obese, and 97% of Aboriginal adults do not consume the recommended daily amounts of fruit and/or vegetables [2]. The causes of the health gap between Aboriginal and non-Aboriginal people are complex and include a range of social, cultural and environmental factors. Addressing health inequities and closing the life expectancy gap are key priorities for Aboriginal and non-Aboriginal organizations in Australia, including national and state governments [3].

There is strong evidence that lifestyle interventions addressing physical activity and diet are effective in promoting behavioural change [4], reducing chronic disease risk factors [5], and preventing chronic disease [6, 7] in the general population. A range of interventions to improve behavioural risk factors for chronic disease have been implemented in Aboriginal populations in Australia. Some programs have found short-term health improvements such as increased physical activity, improved diet and small decreases in weight, BMI and waist circumference [8–11]. For example, the Aboriginal and Torres Strait Islander Women’s Fitness Program was a structured, 12-week group program aimed at reducing waist circumference and improving metabolic health. The program was implemented in an urban area, and included twice-weekly exercise classes and four nutrition workshops. Compared with waitlisted controls, participants showed modest reductions in weight, BMI and blood pressure, which were maintained at three month follow-up [12]. Another program, a 12-week exercise intervention implemented with Aboriginal men in a regional area, resulted in significant reductions in BMI and waist circumference compared to controls. However, the small sample size (16 participants, 10 controls) is a significant limitation of this study [13].

Despite the growing evidence for effective community-based lifestyle interventions for Aboriginal people, previous studies have been conducted in a single Aboriginal community, and to date there have been no evaluations of this type of program implemented on a larger scale in diverse Aboriginal communities. Since 2012, New South Wales (NSW) Health has partnered with NSW Rugby League to implement the NSW Knockout Health Challenge (the Challenge), a healthy lifestyle program for Aboriginal communities in NSW. To build the evidence base for large-scale interventions to promote healthy lifestyles in Aboriginal communities, an evaluation was undertaken to assess the impact of the 2013 Challenge on weight loss and health behaviour change among participants in a number of metropolitan, regional and rural Aboriginal communities in NSW.

## Methods

### Intervention – the NSW Knockout Health Challenge

The Challenge is a state-wide, community-led weight loss and healthy lifestyle program, in which teams compete to achieve the greatest weight loss over a 16-week period. The Challenge has been implemented annually since 2012, and is open to teams of Aboriginal people from across NSW. The aim of the Challenge is to promote weight loss and healthy living through increased physical activity and improved nutrition. In 2013, 22 teams from across NSW took part in the Challenge. The main components of the Challenge in 2013 were:

1. Establishment of the Knockout Challenge Town Committee and Team: Town Committees were established by communities interested in participating in the Challenge. Town Committees were made up of local volunteers from the health, local government, Aboriginal and community sectors, such as Aboriginal Community Controlled Health Services, Aboriginal Land Councils, Medicare Locals and Local Health Districts (LHDs). The Town Committees promoted the Challenge within their community, invited community members to join the Challenge team via targeted invitations and community-wide advertisement, and assisted Challenge teams to link with local health services and other community organisations. Challenge teams comprised 20 to 30 people and were led by a team manager whose role was to coordinate team activities.
2. Participation in the Challenge period: In 2013, the Challenge ran from March to June. During the Challenge, teams competed to achieve the greatest weight loss over a 16-week period. Teams developed their own healthy lifestyle activities to suit their local needs and resources. Although activities varied across the teams, teams typically participated in a diverse range of activities such as group fitness training, gym sessions, cooking workshops, weekly team meetings, monthly weigh-ins, visits to health care providers (e.g. general practitioner, Aboriginal Medical Service, nurse) to receive individual health advice, and dietitian consultations. Participants were also able to join the NSW Get Healthy Information and Coaching Service (a free telephone service staffed by qualified health coaches to support adults to make lifestyle changes) [14], and received motivational messages and support from Aboriginal National Rugby League players via Facebook. Challenge activities were supported by the state-wide implementation team, who provided training for team managers to develop programs of activities for their teams. Teams also received $1000 start-up grants to support Challenge activities (e.g. to purchase exercise equipment), and were encouraged to link with local health organisations to receive ongoing local support. At the end of the Challenge, the three teams with the greatest weight loss percentage received a funding grant to further promote healthy lifestyles in their community; with grants of $20,000, $10,000 and $5,000 for first, second and third place respectively.
3. Participation in the maintenance period: The 2013 Challenge was followed by a 12-week maintenance period (July to September 2013), in which teams were encouraged to stay together and maintain weight loss and lifestyle changes. During this period, Challenge teams had the opportunity to run a pedometer challenge where participants competed to achieve the greatest step count, and to compete in the Challenge Carnival, a sports day in which Challenge teams competed against each other in a range of sports and fitness activities.

### Evaluation methods

The evaluation of the Challenge was completed between March 2013 and March 2014, and had three components: 1) quantitative assessment of participants’ body composition and health behaviours, 2) quantitative and qualitative assessment of participants’ experiences of the Challenge, and 3) qualitative interviews with team managers and the state-wide implementation team. This paper presents findings for components 1 and 2.

#### Quantitative data on body composition and health behaviours

All participants were invited to provide body composition and health behaviour data at four time points: at the start of the Challenge (March 2013, T1), the end of the Challenge (June 2013, T2), 5 months after completion of the Challenge (November 2013, T3) (selected to take place after a significant community event, the NSW Aboriginal Rugby League Knockout Carnival which was held in October), and 9 months after completion of the Challenge (March 2014, T4) (selected to coincide with the registration period for the 2014 Challenge).

##### Body composition

At T1 and T2, participants’ weight, height and waist circumference were objectively measured by a health professional; this was performed by their usual doctor or nurse. At T3, participants were invited (and systematically reminded) to attend a health professional to have their weight and waist circumference objectively measured, and provided a shopping voucher as reimbursement for their time. The intended evaluation design was to repeat the objective measurement of weight at T4, however this was not conducted due to a low response rate to the objective measures at T3.

##### Health behaviours

At T1 and T2, participants completed a standard paper form collecting data on age, gender, chronic disease diagnoses, and self-reported health behaviours (fruit and vegetable consumption and physical activity levels). All self-reported measures were previously validated and are widely used to measure health behaviour [15, 16]*.* At T3 and T4, the self-report measures were collected via computer assisted telephone interview (hereafter “telephone survey”), utilising the same questions as the T1 and T2 paper forms. In anticipation that the response rate to the T3 body composition measurement may be low, participants were also invited to self-report their current weight as part of the telephone survey at both T3 and T4 (participants were asked “What is your current weight?”).

#### Quantitative and qualitative assessment of participants’ experiences of the Challenge

In the T3 and T4 telephone surveys, participants were asked which Challenge activities they took part in, and perceived personal and community impacts of participating in the Challenge. For each of these questions, participants were read a list of potential options, and asked to identify which options applied to them (Yes/No response, multiple responses allowed). Participants were also asked an open-ended question about the personal and community impacts of the Challenge.

### Statistical analyses

#### Quantitative data on body composition and health behaviours

The data were cleaned to remove duplicate records and outliers. Outliers were defined as over 30% change in weight between any two consecutive time points; two outliers were removed – one at T3 and one at T4.

Baseline demographic characteristics of participants who provided data at both T1 and T2 were compared with participants who provided data at T1 only, using t-tests for means and McNemar’s chi-squared tests for proportions. Physical activity levels, fruit intake and vegetable intake were compared with Australian national guidelines [17, 18].

Two separate sets of analyses were completed to assess program impact. The first set of analyses comprised participants with data from all four time points. Body composition and health behaviours were compared for the following pairs of time points: T1-T2, T1-T3, and T1-T4. Changes in continuous variables between time points were analysed using linear mixed models. Included in the models were gender (as a fixed factor) and team (as a random factor). The second set of analyses repeated these procedures for participants who had data at each pair of time points (T1-T2, T1-T3, T1-T4).

In addition, to identify which components of the Challenge were associated with the greatest weight loss, the relationship between BMI change from T1 to T2 and participation in different components of the program was tested by multiple regression using forced entry. Factors included in the model were participation in various components of the Challenge (monthly weigh-ins, team meetings, accessing the Challenge Facebook page, visits to health care providers as part of the Challenge, dietitian consultations, contact with the Get Healthy Service during the Challenge, frequency of team training, and frequency of individual training). Demographic characteristics (age, gender, team, number of chronic illnesses, weight at T1, and whether the participant took part in the 2012 Challenge), were included as potential confounders.

All models satisfied the assumptions of lack of auto correlation, and linearity for metric predictors; variation inflation factor and tolerance statistics were within acceptable ranges. Statistical significance was assessed at *p*=0.05 in all analyses. All quantitative analysis was completed using SPSS V22.

#### Quantitative and qualitative assessment of participants’ experiences of the Challenge

For quantitative data, the proportion of participants responding “Yes” to each response option was calculated. Responses to the open-ended question were transcribed, and analysed using a thematic analysis approach [19]. Analysis was conducted by one researcher, with review by a second researcher. The analysis of the open-ended telephone survey question involved identifying major themes of the impacts, whether these were negative or positive, and the type of impact (whether the changes were in knowledge, attitudes, behaviours, or health and social outcomes).

## Results

### Participation in the Challenge and evaluation

In 2013, 586 people registered for the Challenge in 22 teams. Teams were from 19 Aboriginal communities across NSW - 14 in regional or rural areas and 5 in metropolitan areas. The number of participants per team ranged from 22 to 30 (mean=26.6). The number of participants who provided data varied for each time point: T1 (*n*=575, 98.1%), T2 (*n*=377, 64.3%), T3 via telephone survey (*n*=271, 46.2%) and objective measurement of weight and waist circumference by a health professional (*n*=76, 13.0%), and T4 via telephone survey (*n*=195, 33.3%). T3 and T4 weight data presented below are self-reported data from the T3 and T4 telephone surveys. Objective weight data collected at T3 were excluded from analysis due to the low response rate. After excluding the T3 objective weight data, twenty-one per-cent of participants (*n*=122) provided data at all four time points (based on objective weight data at T1 and T2, and self-report weight data at T3 and T4).

### Profile of Challenge participants

The mean age of participants at the start of the Challenge was 39 years (SD=13.6 years, *n*=575), ranging from 18 to 82 years. The majority of participants were women (72%). Twenty-one per cent of participants in the 2013 Challenge had also taken part in the 2012 Challenge. At commencement the mean weight of participants was 98.5kg (SD=22.4kg, *n*=575), mean BMI was 35.7kg/m^2^ (SD=7.8 kg/m^2^, *n*=575) and mean waist circumference was 113.3cm (SD=16.8cm) (Table 1). Forty-one per cent of participants had a previous diagnosis of, and/or were being treated for, a health condition associated with body composition, physical activity and diet. Baseline characteristics between participants who provided data at T1 and T2 (*n*=377) were similar to characteristics of participants who provided data at T1 only (*n*=199); the only statistically significant difference was that participants who provided data at T1 only were more likely to consume the recommended amount of vegetables per day (*p*=0.004) (Table 2).

**Table 1:** Summary of body composition and health behaviour for participants at each time point of the Knockout Health Challenge 2013

Measure	Measure at start of Challenge (T1)		Change at subsequent time points compared with T1					
	N	T1 (SD)	N	T1-T2 (95%CI)	N	T1-T3 (95% CI)	N	T1-T4 (95% CI)
Mean weight (kg)	575	98.5 (22.4)	377	-2.3* (-2.8, -1.8)	252	-4.6* (-5.5,-3.6)	184	-2.4* (-3.4, -1.3)
Mean BMI (kg/m^2^)	575	35.7 (7.8)	377	-0.8* (-1.0, -0.7)	252	-1.6* (-2.0, -1.3)	185	-0.9* (-1.3, -0.4)
Mean waist circumference (cm)	575	113.3 (16.8)	352	-3.7* (-4.5, -3.0)				
Mean number days per week active for 30mins or more	545	2.0 (2.0)	312	+0.8* (0.5, 1.1)	266	+0.6* (0.3, 0.9)	192	+0.6* (0.3,0.9)
% respondents sufficiently active	545	12.3 (1.0)	312	+6.5* (4.0, 9.7)	266	+7.9* (5, 11.8)	192	+6.3* (3.3, 10.7)
Mean number serves of vegetables per day	545	2.3 (1.5)	312	+0.5* (0.4, 0.7)	268	+0.3* (0.1, 0.5)	193	+0.1 (-0.1, 0.4)
% eat recommended daily serves of vegetables	545	8.4 (1.0)	312	+7.0* (4.5, 10.5)	268	+3.7 (1.8, 6.8)	193	-0.5 (-2.1, 0.0)
Mean number serves of fruit per day	544	1.5 (1.2)	313	+0.4* (0.2, 0.6)	267	+0.5* (0.4, 0.7)	193	+0.4* (0.2,0.6)
% eat recommended daily serves of fruit	544	47.4 (2.0)	312	+15.0* (11.2, 19.5)	268	+26.2* (21.0, 31.9)	193	+20.2* (14.8,26.6)

*p<0.05

**Table 2:** Comparison of baseline characteristics of participants who provided data at T1 and T2 (*n*=377) and those who provided data at T1 only (*n*=199)

	Participants with data at T1 and T2 *N*=377	Participants with data at T1 but not T2 *N*=199
Mean age (years)	40 years	38 years
Female (%)	74.0	69.3
Overweight (BMI>= 25.0kg/m^2^) (%)	94.7	93.5
Type 2 diabetes (%)	16.1	16.8
High cholesterol (%)	15.9	14.4
High blood pressure (%)	23.7	27.5
Unstable asthma (%)	5.6	6.0
Inadequate daily fruit consumption (%)	50.9	47.2
Inadequate daily vegetable consumption (%)	91.0	82.9*
Daily smoker (%)	27.3	25.1

*p<0.05

### Participation in the Challenge

In the T3 telephone survey (*n*=271), participants were asked what types of activities they participated in during the Challenge, and how often. Participants were also asked whether they participated in the maintenance phase activities (pedometer challenge and Challenge Carnival). The activities participants most commonly took part in were monthly weigh-ins (87%), team exercise sessions (86%), individual exercise sessions (83%), visits to health care providers (78%) and team meetings (73%). Participants reported they were involved in an average of 4 different weight loss or healthy lifestyle activities during the Challenge (SD=1.5). Sixty-three per cent of participants reported participating in team exercise sessions at least once per week, and 73% reported participating individual exercise sessions at least once per week. In the maintenance period, 20% of participants took part in the pedometer challenge, and 30% took part in the Challenge carnival.

### Impact on weight and health behaviour

The results presented below are for participants who provided data for all four time points (*n*=122) (Table 3). There was a similar pattern of results for participants who provided data at each pair of time points (Table 1).

**Table 3:** Summary of body composition and health behaviour for participants with data at all four time points of the Knockout Health Challenge 2013

Measure	N	Measure at start of Challenge T1 (SD)	Change at subsequent time points compared with T1		
			T1-T2 (95% CI)	T1-T3 (95% CI)	T1-T4 (95% CI)
Mean weight (kg)	122	98.0 (20.2)	-2.3* (-3.0,-1.9)	-4.6* (-5.5,-3.6)	-2.3* (-3.3,-1.3)
Mean BMI (kg/m^2^)	122	35.8 (6.7)	-0.9* (-1.0, -0.7)	-1.6* (-2.0, -1.3)	-0.8* (-1.2, -0.4)
Mean waist circumference (cm)	352	112.1 (15.5)	-3.7* (-4.5, -3.0)		
Mean number days per week active for 30mins or more	109	2.0 (1.8)	+0.9* (0.4, 1.3)	+ 0.7* (0.3, 1.1)	+0.4 (-0.0, 0.9)
% respondents sufficiently active	109	10.1 (3.0)	+6.4 (2.6, 12.8)	+10.1* (5.1, 17.3)	+4.6 (1.5, 10.4)
Mean number serves of vegetables per day	109	2.6 (1.4)	+0.4* (0.1, 0.7)	+0.1 (-0.2, 0.4)	-0.1 (-0.4, 0.2)
% eat recommended daily serves of vegetables	109	9.2 (3.0)	+6.4 (2.6, 12.8)	+5.5 (2, 11.6)	-2.8 (-7.8, -0.6)
Mean number serves of fruit per day	109	1.6 (1.0)	+0.3* (0.1, 0.6)	+0.4* (0.1, 0.6)	+0.3* (0.1, 0.6)
% eat recommended daily serves of fruit	109	49.5 (5.0)	+20.2* (13.1, 28.9)	+21.1* (13.9, 30.0)	+18.4* (11.6, 26.9)

*p<0.05

#### Body composition

From T1 to T2, participants reduced their weight by an average of 2.3kg (95%CI -3.0 to -1.9, *p*<0.001), reduced their waist circumference by 3.7cm (95%CI -4.5 to -3.0, *p*<0.001) and reduced their BMI by 0.9kg/m^2^ (95%CI -1.0 to -0.7, *P*<0.001). Based on self-reported weight data from T3 and T4, these changes in body composition were maintained at T3 and T4. Overall, mean weight loss from T1 to T4 was 2.3kg (95%CI -3.3 to -1.3, *p*<0.001).

#### Physical activity

From T1 to T2, the self-reported mean number of days participants were physically active for 30 minutes or more increased by 0.9 days per week (95%CI 0.4 to 1.3, *p*<0.001), from 2.0 to 2.9 days per week. At T4, the mean number of active days remained higher than at T1, but was not statistically significant. The proportion of participants meeting the Australian physical activity recommendations did not change significantly from T1 to T2, but increased significantly from T1 to T3 (*p*<0.05), however this was not maintained at T4.

#### Diet

From T1 to T2, the self-reported mean number of servings of fruit per day increased significantly by 0.3 serves (95%CI 0.1 to 0.6, *p* = 0.016) from 1.6 to 1.9 serves. This was maintained at T3 and T4. Similarly, the proportion of participants consuming the recommended daily serves of fruit increased significantly by 20.2% (95%CI 13.1 to 28.9, *p*=0.002), from 49.5% to 69.7%. This was maintained at T3 and T4.

From T1 to T2, the self-reported mean number of daily servings of vegetables increased significantly by 0.4 serves (95%CI 0.1 to 0.7, *p*=0.007), however this was not maintained at T3 or T4. There was no significant change in the proportion of participants consuming the recommended daily serves of vegetables.

#### Factors associated with BMI reduction

For all participants who provided data at T1 and T2 (*n*=192), female participants (*p*=0.008), those who engaged in more team training sessions (*p*=0.025), and those who were heavier at the outset (*p*=0.002) were more likely to reduce their BMI from T1 to T2 (Table 4).

**Table 4:** Participation and demographic factors associated with BMI change for participants from T1 to T2 of the Knockout Health Challenge 2013 (*n*=192)

Variable	B	95% CI	SE B	β	*p*
(Constant)	1.115	(-0.638, 2.867)	.888		.211
Participation in monthly weigh-ins	.694	(-0.333, 1.722)	.521	.105	.184
Participation in team meetings	-.270	(-0.97, 0.431)	.355	-.058	.448
Access to the Challenge Facebook page	-.224	(-0.76, 0.312)	.272	-.061	.410
Visits to health care providers (general practitioner, Aboriginal medical service, nurse) as part of the Challenge	-.144	(-0.872, 0.584)	.369	-.030	.697
Dietitian consultations	.223	(-0.323, 0.768)	.276	.059	.421
Participation in the 2012 Challenge	-.447	(-1.069, 0.175)	.315	-.105	.158
Contacted Get Healthy Service during the Challenge	.065	(-0.722, 0.852)	.399	.012	.871
Frequency of team training	-.211	(-0.395, -0.027)	.093	-.173	.025*
Frequency of individual training	.077	(-0.067, 0.222)	.073	.075	.293
Number of chronic illnesses	.133	(-0.146, 0.413)	.142	.082	.348
Weight at start of Challenge	-.022	(-0.036, -0.008)	.007	-.237	.002*
Female	-.858	(-1.491, -0.225)	.321	-.200	.008*
Age	-.020	(-0.045, 0.004)	.012	-.145	.104
Team	-.025	(-0.069, 0.018)	.022	-.088	.251

*p<0.05

#### Other impacts of the Challenge

In the T3 telephone interviews, participants were asked to describe the impacts of participating in the Challenge at personal and community level. Responses to the list of potential impacts, and illustrative quotes from responses to the open-ended question, are presented below.

Participants identified a range of benefits of participation in the Challenge. Ninety-one per cent of participants felt that participation in the Challenge had improved their physical health. Other benefits included improved knowledge of nutrition and physical activity (90%), adopting a healthier diet (90%), and increased physical activity (88%).

*I started eating healthier and thinking about what I am eating. Instead of grabbing a can of drink, I am going for water instead now.*

*Just a better awareness of diet and exercise. It’s important.*

Many participants also reported improvements in wellbeing (76%) and reduced anxiety and stress (76%).

*It helped me improve my self-esteem to get motivated to start exercising.*

*Better cholesterol, sugar dropped dramatically and generally my overall wellbeing mentally improved.*

Participants also identified community benefits including improved awareness of healthy lifestyles in the community (88%), and increased community pride and connectedness (87%). Very few participants reported any adverse impacts from being involved in the Challenge, however some identified negative impacts such as community issues (8%) and being frustrated when other team members dropped out of the Challenge (5%). Many participants planned to take part in the next Challenge, with 44% of participants reporting they had re-enrolled for the 2014 Challenge.

## Discussion

To our knowledge this is the first time a state-wide program targeting overweight and obesity among Aboriginal people has been successfully implemented and evaluated in a large number of communities. Our results show that Challenge participants lost weight and reduced their BMI, and these changes were maintained 9 months after the Challenge. Participants also increased their physical activity and mean daily fruit and vegetable intake during the Challenge; the increase in physical activity was maintained at 5 months, and fruit intake was maintained at 9 months, but vegetable intake was not maintained after the Challenge. Participants also identified a range of other benefits of participation, such as improved wellbeing.

At the start of the Challenge most participants were overweight, and only a small proportion of participants were meeting health recommendations for physical activity and diet. In addition, the proportion of participants who were overweight and physically inactive was substantially higher than the general NSW Aboriginal population [20]. The health profile of participants suggests the program successfully reached people at high risk of chronic health conditions.

The average weight loss observed in our study (mean weight loss of 2.3kg from the start to the end of the Challenge) was greater than that reported in other similar intervention studies with Aboriginal people, which have reported weight loss of 1.3 to 1.7kg [10, 12, 13]. A significant finding of our study is that participants’ weight loss was maintained after the Challenge. Previous intervention studies with Aboriginal people have either not conducted long-term follow up [9, 10, 12, 13], or found that weight loss was not maintained [11]. In addition, research in the general population suggests participants in lifestyle interventions typically regain some weight in the following year [21]. Although participants’ mean weight loss was modest, these changes are likely to have had a positive impact on participants’ health. From the start of the Challenge to 9 months after, participants’ mean weight improved from class 2 obese (which is associated with a severe risk of comorbidities [22]) to class 1 obese (moderate risk of comorbidities). There is evidence that even modest weight loss can result in positive metabolic changes for individuals [23].

A key strength of the Challenge model is that it was led by local Aboriginal communities and participants were local Aboriginal people. Community engagement in program design and implementation is considered a critical determinant of program effectiveness of healthy lifestyle programs for Aboriginal people [11, 24], and is widely acknowledged in the literature [25–27], by government [3] and by Aboriginal communities [28] as key to closing the gap in health outcomes between Aboriginal and non-Aboriginal people. Another strength of the Challenge model is the flexibility to adapt the program to suit local contexts. The program was devised to be accessible for participants across NSW, with a large majority of teams from regional or rural areas. This highlights the potential for the Challenge model to be adapted to have a broader reach to Aboriginal populations across NSW and Australia, while maintaining flexibility to address health issues that are local priorities. In addition, the finding that 21% of participants in the 2013 Challenge had also participated in the 2012 Challenge suggests the potential for the Challenge to become an annual event for some participants and communities.

The study has identified areas in which the Challenge model could be strengthened. Firstly, men were less likely than women to participate in the Challenge, and less likely than women to reduce their BMI during the Challenge. Low participation of men is not unique to the Challenge [29], with men’s health seeking behaviours substantially lower than women’s among both Aboriginal [30] and non-Aboriginal populations [31, 32]. Future research may seek to identify barriers to participation in healthy lifestyle interventions such as the Challenge, and whether these differ between Aboriginal men and women. Measures to increase male participation in future Challenges warrant further exploration, and could include strategies such as engaging male community leaders, and ensuring teams have access to both male and female health professionals [33]. Second, further efforts are required to support participants to increase vegetable intake. Evidence for effective interventions to increase individuals’ vegetable intake in Aboriginal populations is limited [34], however interventions such as brief individual behavioural counselling [35] have been found to be effective in non-Aboriginal populations and could feasibly be incorporated in the Challenge model.

The evaluation has several limitations. First, weight was objectively measured at the start and end of the Challenge, but self-reported 5 and 9 months after the Challenge. Due to this change in measurement method, the weight data at 5 and 9 months must be interpreted with caution. Attempts were made to objectively measure weight at 5 months, however the low response rate meant that a more pragmatic approach had to be adopted. Given that changes in self-reported physical activity and diet were maintained at 5 and 9 months, it is reasonable to expect that participants would also continue to lose weight. Second, there was loss to follow-up over the course of the evaluation, with 46% of participants followed up at 5 months, and 33% at 9 months. However, the response rates are similar to other surveys of Aboriginal people, such as the response rate to the biomedical component of the combined National Aboriginal and Torres Strait Islander Health Measures Survey sample (40.4%) [36]. Third, no data were collected on the sociodemographic characteristics of participants, and we are therefore unable to comment on the reach of the program across socioeconomic groups. A final limitation relates to the evaluation design. The evaluation used a pre-post design and did not include a control group. While experimental designs such as randomized controlled trials are preferred to observational designs in most circumstances, they are not always practicable in public health [37] - as was the case for this evaluation. It is increasingly argued that broader interpretations of research designs that place a greater emphasis on context, such as mixed-methods and quasi-experimental designs, are appropriate to answer public health research and evaluation questions [38–40]. Furthermore, evaluations of large scale state-wide interventions like the Challenge are rarely published in the literature [41], highlighting the importance of sharing these findings and lessons learned.

## Conclusions

The Challenge was effective in reducing weight and promoting healthy lifestyles among Aboriginal people in NSW. The Challenge addresses key chronic disease risk factors such as weight and nutrition, and thereby has potential to contribute to closing the gap in health outcomes between Aboriginal and non-Aboriginal people. This evaluation makes a substantial contribution to the evidence base for adaptable healthy lifestyle programs to reduce risk factors for chronic disease among Aboriginal people across Australia.

## Abbreviations

NSW: New South Wales
T1: Start of the Challenge
T2: End of the Challenge
T3: 5 months after completion of the Challenge
T4: 9 months after completion of the Challenge
The Challenge: Knockout Health Challenge
